# Simple surface engineering of polydimethylsiloxane with polydopamine for stabilized mesenchymal stem cell adhesion and multipotency

**DOI:** 10.1038/srep18162

**Published:** 2015-12-09

**Authors:** Yon Jin Chuah, Yi Ting Koh, Kaiyang Lim, Nishanth V. Menon, Yingnan Wu, Yuejun Kang

**Affiliations:** 1 School of Chemical and Biomedical Engineering, Nanyang Technological University, 62 Nanyang Drive, Singapore 637459, Singapore

## Abstract

Polydimethylsiloxane (PDMS) has been extensively exploited to study stem cell physiology in the field of mechanobiology and microfluidic chips due to their transparency, low cost and ease of fabrication. However, its intrinsic high hydrophobicity renders a surface incompatible for prolonged cell adhesion and proliferation. Plasma-treated or protein-coated PDMS shows some improvement but these strategies are often short-lived with either cell aggregates formation or cell sheet dissociation. Recently, chemical functionalization of PDMS surfaces has proved to be able to stabilize long-term culture but the chemicals and procedures involved are not user- and eco-friendly. Herein, we aim to tailor greener and biocompatible PDMS surfaces by developing a one-step bio-inspired polydopamine coating strategy to stabilize long-term bone marrow stromal cell culture on PDMS substrates. Characterization of the polydopamine-coated PDMS surfaces has revealed changes in surface wettability and presence of hydroxyl and secondary amines as compared to uncoated surfaces. These changes in PDMS surface profile contribute to the stability in BMSCs adhesion, proliferation and multipotency. This simple methodology can significantly enhance the biocompatibility of PDMS-based microfluidic devices for long-term cell analysis or mechanobiological studies.

Polydimethylsiloxane (PDMS) silicone elastomer has been receiving much attention as a popular material for developing substrate platforms in mechanobiological^1^,^2^,^3^,^4^ and microfluidic applications^5^,^6^,^7^, owing to its numerous advantages over other fabrication materials. The salient characteristics of PDMS giving rise to wide applications include its tunable elastomeric properties, low cost, gas permeability, optical transparency, low auto fluorescence, nano-scale precision, and easy moldability^8^,^9^,^10^. However, the use of PDMS for cell culture often poses several challenges over long-term studies. The intrinsic high surface hydrophobicity of PDMS has been identified by many studies to be the primary factor that causes poor cell adhesion, creating and dissociating islands of cell aggregates^11^,^12^,^13^. Therefore, it is highly demanded to improve the surface biocompatibility of PDMS to facilitate long-term cell studies.

Earlier attempts to reduce surface hydrophobicity by oxygen plasma treatment have shown to improve cell adhesion but the effect was often short-lived due to hydrophobic recovery^14^,^15^. While extracellular matrix protein coating could potentially enhance cell adhesion and proliferation, cell sheet aggregation and detachment was usually observed after prolonged culture due to protein dissociation^13^,^16^. Recently, PDMS surface functionalization with (3-aminopropyl)triethoxy silane (APTES) with glutaraldehyde as crosslinker and coupled with protein coating has shown to improve cell adhesion, proliferation^16^ and osteogenic differentiation over 3 weeks^13^. Although this surface treatment method is very effective, the process involves time-consuming intermediate steps and the human error accumulated between each step may potentially lead to batch-to-batch inconsistencies. Furthermore, the use of toxic chemicals such as (3-Aminopropyl)triethoxysilane (APTES) and glutaraldehyde poses potential health hazards and generate chemical wastes that are toxic to the environment. Therefore, a simple, environmentally safe and effective surface functionalization strategy is crucial for rendering biocompatible PDMS surfaces for long-term cell investigation on PDMS-based lab-on-a-chip devices.

In recent years, the bio-inspired polydopamine (PD) has been widely utilized in the areas of energy, environmental and biomedical sciences^17^. Dopamine undergoes oxidative polymerization in alkaline conditions and has a strong adsorption onto a wide variety of substrates through covalent bonding and strong intermolecular interactions^18^,^19^. Investigations have shown that PD coating reduces substrate surface hydrophobicity^19^ and promotes *in-vitro* tissue development^20^,^21^,^22^. More importantly, PD has been shown to reduce the *in-vivo* toxicity of implanted biomaterials, and thus recommended as a surface coating reagent for cell studies^23^. Nevertheless, the feasibility of PDMS surface functionalization with PD for long-term stem cell culture remains unclear.

Bone marrow stromal cells (BMSCs) are unspecialized stem cells that originate from the mesoderm with self-renewal and multilineage differentiation ability. Furthermore, BMSCs pose minimal issues with regard to ethical concerns or teratoma formation as compared to other types of stem cells, such as embryonic stem cells and induced pluripotent stem cells^24^. These intrinsic features of BMSCs have made them a promising cell source for stem cell therapy and regenerative medicine, thus increasing the number of clinical trials involving BMSCs^24^. To date, many fundamental understandings of BMSCs still remain vague and require extensive studies to resolve numerous scientific questions raised in the attempts of real applications. The regular studies on the differentiation of BMSCs often span over a duration of at least 3 weeks. However, the native PDMS surface property as described previously has limited the experimental studies of BMSC differentiation in microfluidic lab-on-a-chip devices due to its inability to support long-term cell culture.

Inspired by the numerous advantages of polydopamine, it is of great interest to investigate the efficiency of PDMS substrate functionalization to stabilize long-term cell culture, especially in the field of BMSC-based cell studies. The PD concentration, coating time, the resulting wettability and surface chemistry on the PD-coated PDMS are experimentally characterized to obtain fundamental insights into this surface treatment and its effect on the multipotency and stability of BMSC culture over several weeks (Fig. 1). The PD-based surface modification can be utilized as a simple and efficient alternative to promote long-term BMSC culture while maintaining their multipotency on the PDMS substrates.

## Results and Discussion

### Concentration and time of PD coating

**Figure 2:**
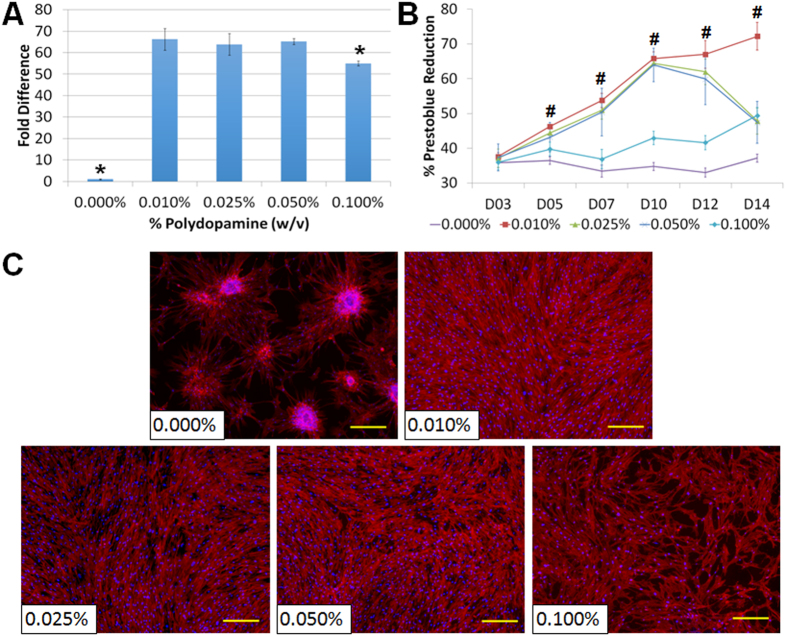
Effect of polydopamine concentration (0-0.100% w/v) on MSCs (A) initial adhesion, (B) proliferation and (C) adhesion stability after 2 weeks. The actin cytoskeleton of BMSCs was stained red (rhodamine phalloidin) and the cell nucleus was stained blue (DAPI), scale bar = 200 μm. *p-value < 0.0132 showed significant differences between any two groups (Tukey HSD test). #p-value < 1 × 10^−4^ revealed significant difference between the groups (One way ANOVA). All data are presented as mean ± s.d. (n = 4).

PD has been recently exploited to improve cell behaviour on various substrates^25^,^26^,^27^,^28^. Nevertheless, the effect of PD concentration and coating time on the stability of BMSC growth remains poorly understood when functionalizing native PDMS substrates, which is vital to develop a surface modification strategy for stable and long-term BMSC culture. To address this important issue, a series of PD concentrations increasing from 0 to 1.0% (w/v) was coated on the native PDMS substrates for 24 h and assessed for the initial cell adhesion as well as the prolonged cell proliferation. When the native PDMS surface was coated with PD at various concentrations, we observed at least a 40-fold increase in cell adhesion (p-value = 6 × 10^−14^, one way ANOVA) as compared to the uncoated PDMS surfaces (Fig. 2A and Supplementary Fig. S1A), which was a strong evidence suggesting that the presence of PD facilitated initial cell adhesion. On the native uncoated PDMS surface, cell population did not increase in 2 weeks (Fig. 2B) and islands of cell clumps were observed in the culture (Fig. 2C). Similarly, cell proliferation on PDMS coated with more than 0.1% (w/v) PD was not optimal (Supplementary Fig. S1B). Although the cell proliferation was enhanced on PDMS surfaces coated with 0.025–0.05% (w/v) PD (p value = 1 × 10^−4^, one way ANOVA) for the first 10 days, this promoting effect was however short-lived showing decrease in cell population from Day 11 onwards (Fig. 2B and Supplementary Fig S1B). Live-Dead Staining revealed minimal cell death on all PD-coated PDMS (Supplementary Fig. S2), suggesting that the PD coating was not toxic to the BMSCs. This in turn implied that the reduction of cell proliferation after Day 10 might be related to unstable cell adhesion. The decrease in cell population on PDMS coated with 0.025% and 0.050% (w/v) PD was further confirmed by microscopic imaging, which revealed sites of cell aggregation and peeling-off upon confluence (Fig. 2C and Supplementary Fig. S3). On the other hand, the cell population on PDMS coated with more than 0.100% (w/v) PD did not reach confluence (Fig. 2C and Supplementary Fig. S3) after 2 weeks of culture. These results hereby indicated that PD coating concentrations higher than 0.025% (w/v) were not able to maintain a stable long-term BMSC culture on the PDMS surface. Amongst the various concentrations studied, only 0.01% (w/v) PD-coated PDMS surface exhibited continuous enhancement to the cell proliferation (Fig. 2B, p-value < 1 × 10^−4^, one way ANOVA) with stabilized and confluent BMSC cell sheet for over 2 weeks (Fig. 2C).

**Figure 3:**
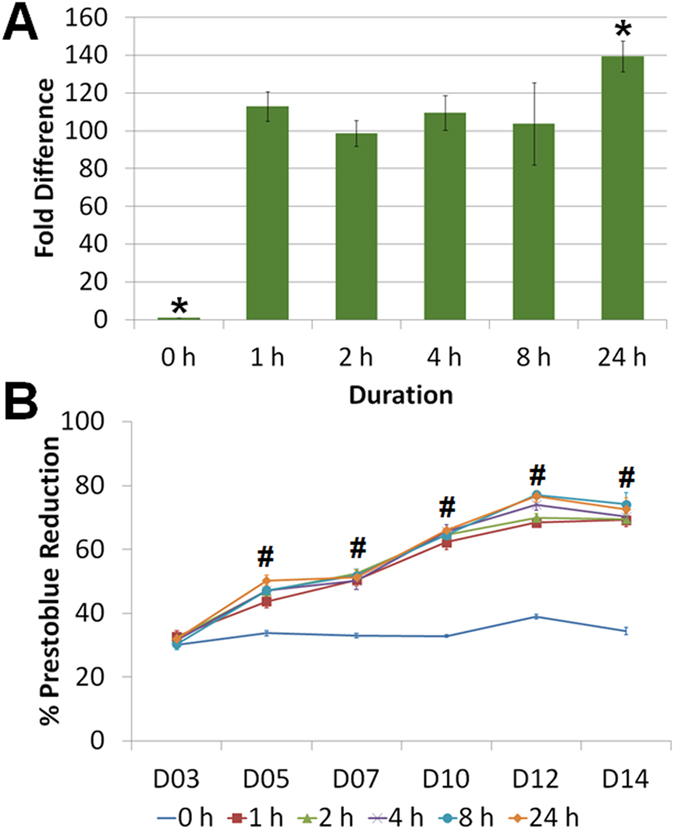
Effect of polydopamine (0.01% w/v) coating time (0−24 h) on MSC (A) initial adhesion and (B) proliferation. *p-value < 0.0419 shows significant differences between any two groups (Tukey HSD test). #p-value < 1.35 × 10^−9^ reveals significant difference between the groups (One way ANOVA). All data are presented as mean ± s.d. (n = 4).

The coating time of 0.01% (w/v) PD varied from 0 to 24 h to investigate its effect on both BMSC adhesion and proliferation. The results indicated at least 80-fold increase in initial cell adhesion as compared to native PDMS (p-value = 5.45 × 10^−10^, one way ANOVA) if the coating time exceeded 1 h (Fig. 3A). Although the highest cell adhesion was observed on the PDMS with prolonged PD coating of 24 h, faster coating between 1 to 8 h did not show significantly different effects. Interestingly, the long-term BMSC proliferation rate over 2 weeks was promoted on all PD-coated PDMS while not sensitive to the coating time (Fig. 3B, p-value = 1.35 × 10^−9^, one way ANOVA).

### PD coating in combination with collagen coating

**Figure 4:**
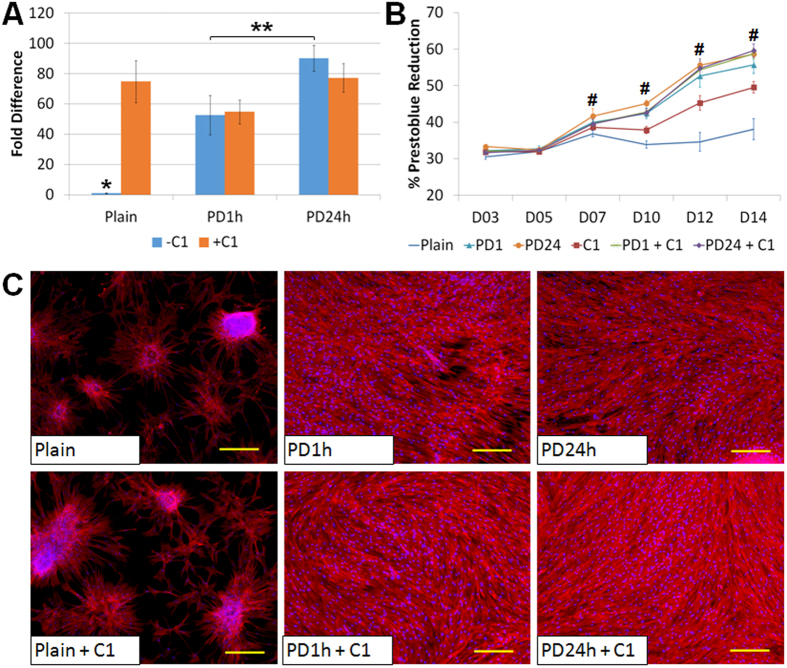
Effect of polydopamine coating with collagen on MSCs (A) initial adhesion, (B) proliferation and (C) adhesion stability after 2 weeks. The actin cytoskeleton of BMSCs was stained red (rhodamine phalloidin) and the cell nucleus was stained blue (DAPI), scale bar = 200 μm. *p-value = 0.00101 showed significant differences between any two groups (Tukey HSD test, n = 4). **p-value = 0.00175 shows significant difference between the groups (Tukey HSD test, n = 4). #p-value < 3.67 × 10^−10^ revealed significant difference between the groups (One way ANOVA). All data are presented as mean ± s.d. (n = 4).

The extracellular matrix plays many critical biological roles^29^,^30^,^31^ and has been shown to be involved in cell adhesion^32^,^33^,^34^ and differentiation^29^,^35^. It is very common to coat the substrate with extracellular matrix proteins to provide amicable microenvironment for better cell growth. Collagen is one of the most abundant proteins in the mammalian tissues and has been widely utilized as a coating material to improve the biocompatibility of various culture substrates^29^,^30^,^32^,^33^,^34^,^35^ and the stem cell differentiation^29^,^35^. Nevertheless, conventional collagen coating by physical adsorption on PDMS surfaces was not optimistic in supporting long-term cell culture due to formation of cell clusters and cell sheet dissociation after confluence^13^,^16^. This was further confirmed by our experimental observation (Fig. 4). As shown above, PD coating for 1–24 h could improve cell adhesion and proliferation on PDMS (Fig. 3). It is thus essential to investigate if prior surface treatment with PD coating could also enhance the performance of collagen coating on BMSC behaviour. Herein the effect of collagen coating combined with either 1 h or 24 h PD coating on BMSCs was further investigated.

In a control experiment, collagen coating on native PDMS had shown to enhance initial cell adhesion (Fig. 4A). However, the long-term cell growth with collagen coating only was not optimal (Fig. 4B) with formation cell clusters that could cause cell dissociation easily (Fig. 4C). Subsequent collagen coating on PD-coated PDMS significantly improved cell adhesion (p-value = 0.001, Tukey HSD test) and proliferation rate of BMSCs (p-value < 3.67 × 10^−10^, Tukey HSD test) as compared to both native PDMS and collagen-coated PDMS (Fig. 4), although the PD coating time did not show notable different effect between 1 h and 24 h. These results suggested that PD coating was compatible and could be used in combination with popular surface functionalization with matrix proteins.

### Surface characterization

**Figure 5:**
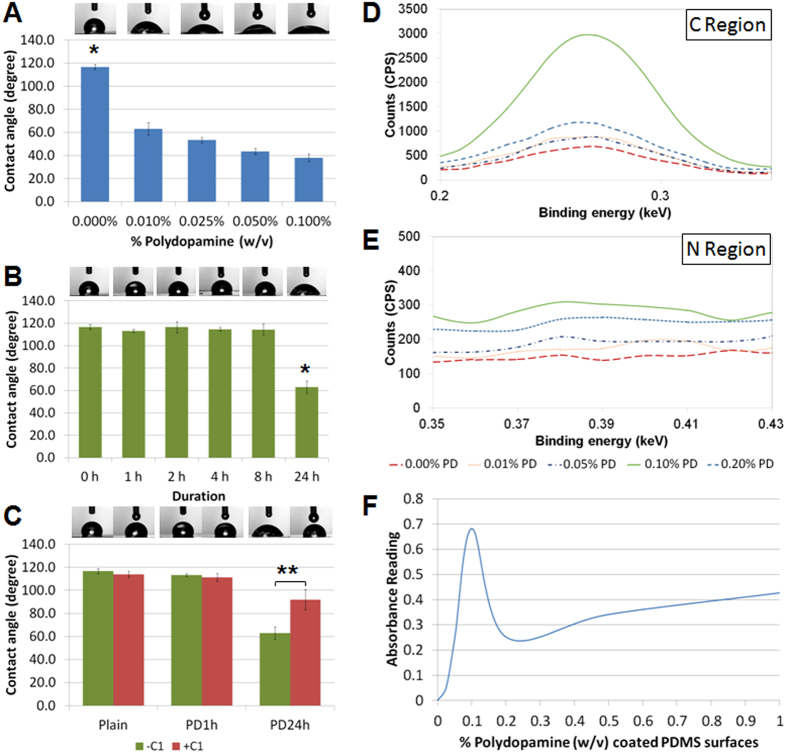
Water contact angle measurement of uncoated/coated PDMS surfaces with (A) different PD concentration after 24 h, (B) different coating time of 0.010% PD and (C) PD and collagen coating. EDS spectrum of uncoated/coated PDMS surfaces for the detection of (**D**) carbon and (**E**) nitrogen elements. (**F**) Ninhydrin assay for the detection of free amine group across different PD concentration coating after 24 h. *p-value < 0.00621 showed significant differences between any two groups (Tukey HSD test). **p-value = 0.00101 showed significant differences between two groups (Tukey HSD test). All data are presented as mean ± s.d. (n = 4).

The cell-surface interaction plays an important role in mediating BMSC adhesion, proliferation and differentiation^36^,^37^,^38^, which may explain the improved MSC behaviour on the PD-coated PDMS observed above. Thus, it is imperative to characterize the surface properties to gain fundamental insights on the cell-surface interaction. There are numerous studies highlighting the inherent hydrophobicity of PDMS as one of the major factors to cause poor maintenance of stable cell culture^11^,^12^,^13^. Modifying the hydrophobic surfaces to hydrophilic can potentially promote cell adhesion and proliferation. Uncoated PDMS exhibited a water contact angle of 116.7 ± 2.21^o^ (Fig. 5A). Increasing the PD concentration resulted in decreased contact angle (40–70^o^, p value < 0.001, Tukey HSD test) on the coated PDMS surfaces (Fig. 5A). All these surfaces had shown to improve initial cell adhesion, while only 0.010% (w/v) PD-coated PDMS surface was able to prolong the stability of BMSC proliferation over 2 weeks (Fig. 2B,C). The study using various coating time with 0.010% (w/v) PD from 1 to 24 h showed that prolonged coating of 24 h significantly reduced the water contact angle (p-value = 0.001, Tukey HSD test) and changed the surfaces from hydrophobic to hydrophilic (Fig. 5B), which could be the major reason that encouraged the initial cell adhesion (Fig. 3A). Interestingly, although 0.010% (w/v) PD coating between 1 to 8 h showed similar hydrophobicity profile as compared to the uncoated PDMS surface (Fig. 5B), all these PD-coated hydrophobic surfaces promoted both BMSC adhesion and proliferation (Fig. 3). These findings suggested that surface wettability might not be a critical factor that contributed to the improved behaviour of BMSCs in this study. The subsequent collagen coating after 24 h PD coating significantly increased the PDMS surface hydrophobicity (Fig. 5C, p-value = 0.001, Tukey HSD test) but did not influence initial BMSC adhesion and proliferation notably (Fig. 4A,B).

Surface chemistry is another major factor that could influence cell behaviour^37^. Thus, we further evaluated the surface chemistry profile of the PD-coated PDMS with energy dispersive X-ray spectroscopy (EDS). The carbon region scan of PD-coated PDMS obtained from EDS revealed higher relative loading of carbon (Fig. 5D), which implicated the successive coating due to the presence of organic PD molecules. As native PDMS lacks the nitrogen atoms, the detection of nitrogen atoms on various PD-coated PDMS further confirmed the existence of PD (Fig. 5E) due to the presence of amine group. Prior studies have suggested that the cell adhesion and growth can be possibly enhanced by the presence of free amine group^39^,^40^, which can be detected by Ninhydrin-based colorimetric assay^41^,^42^. Herein we found a linear relation between the absorbance at 570 nm and the PD concentration (0−0.2%) using ninhydrin reagent for detecting free amine groups in solution (Supplementary Fig. S4). Furthermore, the absorbance analysis on the PD-coated PDMS surfaces under various coating concentrations displayed a distinct peak at 0.100% (w/v) (Fig. 5F) and EDS also verified that the highest amount of nitrogen appeared under this PD coating concentration (Fig. 5E). Particularly, the absorbance varied almost linearly to the PD coating concentration up to 0.1% (Fig. 5F). These results indicated that 0.1% (w/v) coating concentration could maximize the absorption of PD on native PDMS. Considering the previous results, it was implied that the presence of amine groups could generally enhance initial cell adhesion as compared to the native PDMS (Fig. 2A), whereas the stability of BMSC culture was more favourable on surfaces with lower amine contents (Fig. 2B). When the coating time of 0.01% PD was reduced to 1–8 h, the surface hydrophobicity did not change notably (Fig. 5B) while the coated surfaces exhibited similar promoting effect on cell proliferation for long-term BMSC culture (Fig. 3B). These results suggested that the presence of amine groups had predominant effect over surface wettability for the enhanced BMSC adhesion and proliferation.

### BMSC differentiation and multipotency

BMSC differentiation was widely investigated on various substrate platforms for more than 2 weeks^43^,^44^,^45^,^46^. It is vital that the PD-coated PDMS surfaces can maintain the prolonged stability of cells adhesion and capability of BMSC multi-lineage differentiation in long-term induced differentiation culture. The adhesion stability of cell population on the substrates mainly depends on the cell-substrate interaction through adhesins, such as integrins^47^,^48^. To understand the underlying mechanism of the interaction between BMSCs and the PD-coated surfaces, we further analysed the expression of β1-integrin (CD29) and α5-integrin (CD49e) though real time gene expression. The differentiated cells on unmodified surfaces (without collagen and PD coating) were not analysed due to the absence of stabilized cell population. β1-integrin and α5-integrin were observed to be upregulated on most PD-coated PDMS as compared to PDMS simply coated with collagen in both osteogenic and adipogenic induced BMSCs (Supplementary Fig. S5), thus suggesting that the presence of polydopamine could upregulate β1-integrin and α5-integrin to mediate the enhanced cell adhesion on PDMS substrate. Meanwhile, additional coating of collagen on PD-coated PDMS did not show significant influence on the expression of β1-integrin but demonstrated significant upregulation of α5-integrin expression on 24 h PD-coated PDMS (p-value = 0.0395, Tukey HSD test, Supplementary Fig. S5).

**Figure 6:**
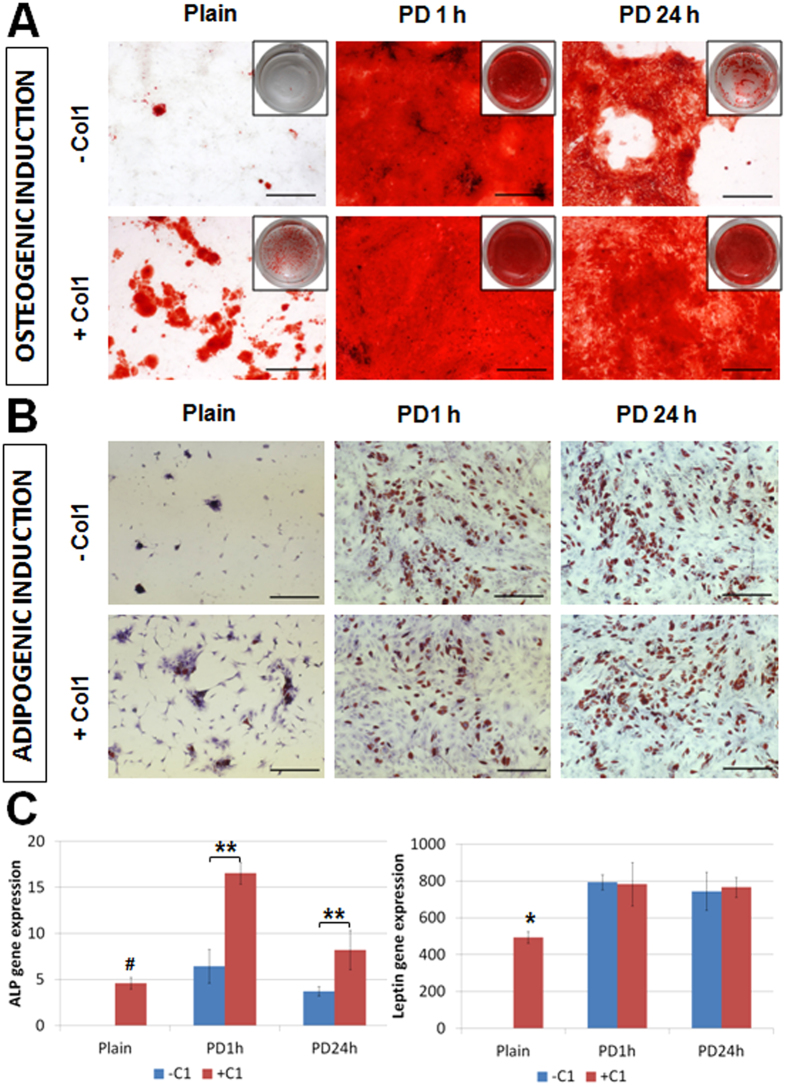
(**A**) Alizarin Red staining, scale = 500 μm and well diameter = 15 mm; (**B**) Oil Red staining, scale bar = 500 μm; (**C**) gene expression of osteogenic (ALP) and adipogenic (Leptin) markers. *p-value < 0.00293 shows significant difference as compared to any other group (Tukey HSD test, n = 4). **p-value < 0.00541 shows significant difference between the groups (Tukey HSD test, n = 4), #p-value < 0.0240 shows significant difference as compared to any PD ± C1 coated PDMS (Tukey HSD test). All data are presented as mean ± s.d. (n = 4).

To further verify the effect of cell adhesion stability on MSC differentiation, MSCs on different PDMS surfaces were subjected to either osteogenic or adipogenic differentiation. Previous studies have demonstrated that increased β1-integrin and α5-integrin can enhance osteogenic and adipogenic differentiations of MSCs through the phosphoinositide-3-kinase (PI3K) signalling pathway^49^,^50^,^51^,^52^. Particularly, the osteogenic capability of MSCs stimulated by PD film could be reduced in the presence of a potent PI3K inhibitor (LY294002)^51^, which further implicated that PD-coated surfaces favoured MSC osteogenesis through the activation of both integrin and PI3K signalling pathways. In the present study, the gene expression of phosphoinositide-3-kinase, catalytic, gamma polypeptide (PI3KCG) within MSCs cultured on PD-coated PDMS surfaces was found to be considerably higher (Supplementary Fig. S5), which was consistent to the prior studies and verified the favoured MSC differentiations through promoted PI3K signalling pathway. Alizarin red staining was used to detect the presence of brick-red nodules, which could indicate the bio-mineralization of BMSCs after 3 weeks of osteogenesis on different surfaces (Fig. 6A). On the other hand, Oil Red O staining was used to identify the accumulation of triglyceride after 3 weeks of adipogenesis, which was presented as red oil droplets within the adipogenic cells (Fig. 6B).

The native PDMS surface was reported to discourage the stabilization of differentiated cell population^13^. This issue was also verified in this study for both osteogenic and adipogenic cell populations (C1− and Plain in Fig. 6A,B). Coating the native PDMS substrates only with collagen type 1 (C1) exhibited minimal positive histological stain in both osteogenic and adipogenic lineages indicating attenuated multipotency of the cell population (C1 + and Plain in Fig. 6A,B) due to their poor adhesion stability (Fig. 4C). In contrast, a layer of stabilized osteogenic or adipogenic cell population was formed after 3 weeks of culture on the PDMS coated with 0.01% (w/v) PD (Fig. 6A,B). A previous study reported that surface amine groups can promote osteogenic lineage^53^, which was consistent with our finding that PD-coated PDMS promoted BMSC osteogenesis considering the abundant amine groups contained in PD. With respect to the PD coating time, although prolonged coating (24 h) enhanced BMSC adhesion and proliferation (Fig. 3) and reduced PDMS hydrophobicity significantly (Fig. 5B,C), the osteogenic lineage exhibited stronger stability (Alizarin Red staining) on the surface under 1 h fast coating (Fig. 6A). These results were consistent to the gene expression by real-time PCR assays, which revealed that although Col1 expression was upregulated on PDMS with either prolonged or fast PD coating (Supplementary Fig. S6), the other major osteogenic marker ALP expression was much higher under fast PD coating (Fig. 6C). It was reported that the hydrophobicity of a biomaterial was able to encourage bone healing by promoting adherence of cells and adsorption of proteins^54^, which might explain our observation that the hydrophobic PD-coated surface (with shorter coating time) facilitated better bio-mineralization. Real-time gene expression analysis further suggested that fast PD coating rendered a PDMS surface that facilitated a higher stabilization of cell adhesion and osteogenic capability through the promotion of α-5 integrin expression and PI3K signalling pathway (p-value < 0.0384, Tukey HSD test, Supplementary Fig. S5).

Meanwhile, there was no much difference in Oil Red staining between 1 h and 24 h PD-coated PDMS (Fig. 6B) and the leptin expression on all PD ± C1 coated PDMS was upregulated as compared to C1-coated PDMS (Fig. 6C) in adipogenic lineage. This implied that both hydrophobic and hydrophilic PD coated PDMS could support adipogenic lineage in long-term culture.

We also investigated the effect of secondary coating with matrix proteins on the BMSC differentiation. It was found that the secondary coating with C1 on PD-coated (0.010% w/v, 24h) PDMS improved the stabilization of osteogenic lineage (Fig. 6A) and increased alkaline phosphatase (ALP) gene expression (Fig. 6C), while having no such promoting effect on the adipogenic lineage (Fig. 6B,C). The differentiated cells on unmodified surfaces were not analysed in the study due to the lack of stabilized cell population. The results suggested that the surface chemistry and hydrophobicity had a combinatorial effect to stabilize and promote BMSC differentiation. Additionally, rapid PD coating of 1 h was sufficient to render a biocompatible PDMS surface for prolonged study of BMSCs.

It is noteworthy that PDMS could adsorb a variety of chemical components introduced by some common supplements (e.g., 10% FBS) to the cell culture medium, such as amino acids, growth factors, vitamins and hormones^55^, which in turn could potentially alter the PDMS surface chemistry and interfere with the PD coating. However, as indicated in this study, the promoting effect of PD coating to the stabilization of cell adhesion, proliferation and multipotency was not affected as compared to the non-coated and collagen-coated PDMS in the presence of FBS-based culture medium. Therefore, these results suggested that the adsorption of a variety of chemical components contained in the culture supplements onto the PDMS substrate did not interfere with the PD coating for stabilized cell culture. Moreover, previous studies have also shown that the use of supplemented culture medium on modified PDMS substrates with (3-aminopropyl)triethoxysilane + glutaraldehyde + C1 did not deteriorate the MSC adhesion, proliferation and differentiation as compared to the commercial tissue culture plates^13^,^16^.

### PD coating for microfluidic chips

There is an increasing demand for long-term stem cell studies using PDMS-based microfluidic systems^56^,^57^,^58^, which however has been limited by the inherent properties of native PDMS. Previously, many microfluidic devices with multi-channel design were used to achieve separation and confinement of different cells types in different compartments for on-chip studies of co-culture, cell migration and other cellular responses^7^,^59^,^60^. As shown above, PD-coated PDMS was able to support long-term BMSC differentiation. Hereby we further investigated if the same surface modification could be applied to an enclosed 3-channel microfluidic system with minimal supply of culture medium, and thus allowing prolonged study of stem cell biology within a microfluidic chip.

**Figure 7:**
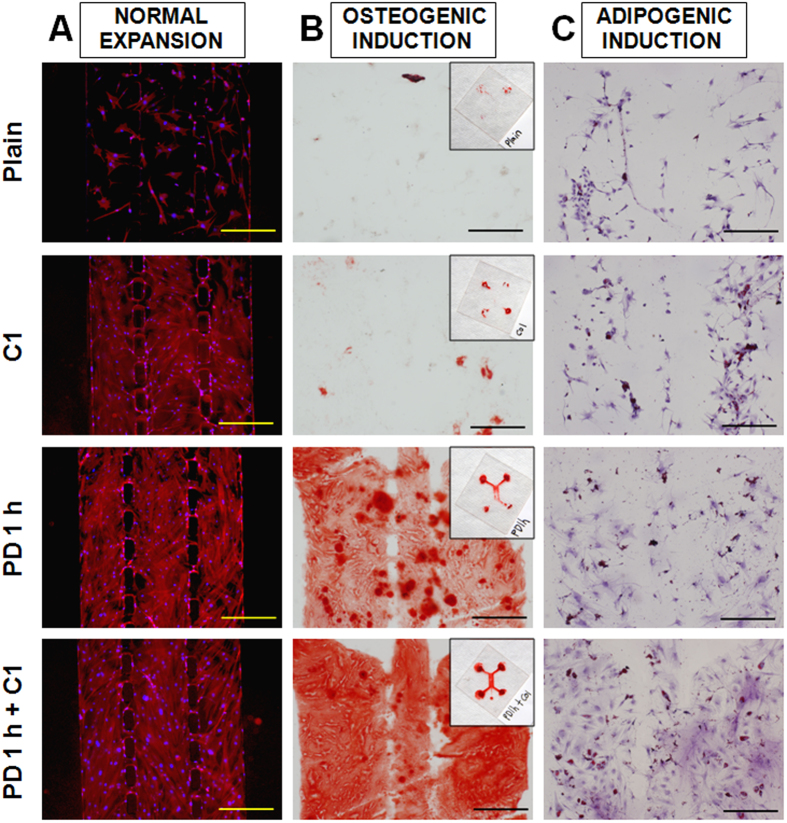
(**A**) F-Actin localization of cells under normal expansion, (**B**) Alizarin Red staining of osteogenic cultures, microfluidic chips = 22 mm × 22 mm, and (**C**) Oil Red staining of adipogenic cultures for 3 weeks in a microfluidic culture system. Scale bar = 500 μm.

Similar to the open PDMS substrates, the native PDMS surface did not encourage the proliferation of BMSCs (Fig. 7A) in the microfluidic system, and thus was unfavourable for both osteogenic and adipogenic differentiations (Fig. 7B,C). While simple collagen coating improved the proliferation of BMSCs in the microfluidic system, histological staining indicated minimal region on the substrate to stabilize osteogenic or adipogenic differentiation of BMSCs. 1 h PD coating further enhanced the BMSC proliferation and notably improved the osteogenesis. Nevertheless, combining PD and collagen coatings rendered a microfluidic system with optimal BMSC proliferation, osteogenic or adipogenic differentiation as compared to single coating with only one of the reagents.

## Conclusions

Surface modification with PD and/or C1 was investigated in this study to improve the biocompatibility of PDMS substrates for long-term BMSC culture. Although increasing the PD coating concentration and time could reduce the hydrophobicity of PDMS, a hydrophobic PDMS surface coated with 0.01% PD for 1 h was shown to induce the optimal enhancement of BMSC adhesion, proliferation, stability and differentiations in long-term culture. These results suggested that modification of PDMS surface chemistry by PD and matrix protein coatings could have predominant effects on the BMSC culture compared to the variation of hydrophobicity. This simple and rapid surface treatment is applicable to open PDMS substrates as well as enclosed PDMS-based microfluidic systems, which can be widely applied in many *in-vitro* studies of cellular physiology, especially in the areas of regenerative medicine and mechanobiology.

## Methods

### Materials

Laboratory chemicals were purchased from Sigma Aldrich (Singapore) and cell culture reagents were purchased from Life Technologies (Singapore) unless stated otherwise.

### PDMS substrate fabrication

PDMS substrates were prepared by mixing ten parts of silicone elastomer base with one part of curing agent (SYLGARD, Dow Corning, USA) and stirred. The mixture was then poured into well plates or culture dishes, degassed for 30 min in a vacuum oven to remove air bubbles, followed by heat-curing at 70 °C for 100 min. Surface modification was performed by immersing the native PDMS surface in dopamine solution (Sigma Aldrich, Singapore) prepared in 10 mM Tris-HCl (pH 8.5). PD concentration (0.000%w/v–0.100%w/v) and duration of coating (0–24 h) were varied to investigate the effect of these parameters on the stability of BMSC adhesion and proliferation. After PD coating, the surfaces were rinsed twice with 1X Phosphate Buffered Saline (PBS) to remove unattached PD molecules. Additional coating of 20 μg/ml collagen type 1 (Life Technologies, Singapore) was performed to evaluate the combinatorial effect of PD and collagen on BMSC behaviour. Lastly, the uncoated and PD-coated PDMS substrates were UV-sterilized for 1 h prior to cell culture.

### Mesenchymal stem cell culture

Human BMSCs were harvested under an IRB-approved protocol as described previously^61^. Primary BMSCs were allowed to grow till confluence in expansion medium comprising low glucose Dulbecco’s modified Eagle medium (DMEM) supplemented with 10% Fetal Bovine Serum (FBS), Penicillin (100 U/ml) and Streptomycin (100 μg/ml) mixture and 2 mM GlutamaxTM at 37 °C in humidified atmosphere with 5% CO_2_. BMSCs of passages 2−4 were used in the experiments for this study.

### Cell adhesion Assay

3000 cells per cm^2^ was seeded and maintained in expansion medium at 37 °C in humidified atmosphere with 5% CO_2_. For cell adhesion assay, samples were collected 1 h after cell seeding, washed twice with 1X PBS to remove non-adherent cells. The adherent cells was frozen at −80 °C for 1 h, thawed and subsequently lysed with a cell lysis buffer comprising 1X CyQUANT GR Dye from the CyQUANT Cell Proliferation Assay Kit (Life Technologies, Singapore) for 5 min. Fluorescence intensity of sample aliquots were measured with Infinite M200 series microplate reader (Tecan, Singapore) under excitation at 485 nm and emission at 535 nm, which reflected the relative amount of DNA in each sample. As the DNA content was constant within each cell, this measurement provided a simple and deterministic means to correlate the total DNA content to the cell numbers and thus provided an accurate quantitation of the adhered cells on the surface. The reading was expressed as a fold difference relative to the fluorescence intensity to the plain PDMS.

### Cell proliferation assay and Fluorescence imaging

The cell proliferation activity of BMSCs on uncoated and coated PDMS surfaces were assessed with PrestoBlue cell viability reagent (Life Technologies, Singapore) according to the manufacturer’s protocol. Briefly, the expansion medium was removed and the cells were washed twice with 1X PBS before incubating with expansion medium containing 10% PrestoBlue reagent for 1 h at 37 °C in humidified atmosphere with 5% CO_2_. Expansion medium containing 10% PrestoBlue reagent in the wells with no cells served as the blank control for this assay. The absorbance of the reduced PrestoBlue reagent for each sample aliquot was read with Infinite M200 series microplate reader under excitation at 570 nm and emission at 600 nm. As the total viable cells correlated with the PrestoBlue dye reduction level, the percentage reduction of the PrestoBlue reagent was calculated according to the manufacturer’s protocol.

To visualize the BMSC populations on different PDMS surfaces, cell cultures after two weeks were fixed with 10% formalin for 20 min, permeabilized with 0.1% Triton X-100 and stained with rhodamine phalloidin (for F-actin localization) and DAPI (for nucleus localization). To assess the viability of the BMSCs on different PDMS surfaces, the unfixed cell population was directly stained with 20 ug/ml fluorescein diacetate (FDA) (Sigma Aldrich, Singapore) and 100 ug/ml propidium iodide (PI) (Sigma, Singapore). Fluorescence images of the cells were captured with an inverted microscope (Olympus IX71, Singapore).

### Surface characterization

Uncoated and PD-coated PDMS surfaces were assessed for wettability and surface chemical composition. All samples were assessed with the same sampling size (n = 4) to ensure the reproducibility of the production process. The water contact angles were measured with a FTA 200 Contact Angle Analyzer (First Ten Angstroms, Virginia, US). The surface chemical composition was analysed using energy dispersive X-ray spectroscopy (EDS). Briefly, PDMS samples were sputter-coated with a layer of platinum and analyzed with EDS (X-Man^N^, Oxford Instruments, Oxfordshire, UK) equipped with JSM-6701F Field Emission Scanning Electron Microscope (JEOL, Singapore). The samples were measured at a magnification of 500X, with beam current and ion energy fixed at 11 μAh and 15 kV respectively. For each sample surface, three different points were scanned and averaged to obtain the atomic composition of carbon, oxygen and nitrogen. Free amine groups were quantified by immersing the uncoated and PD-coated PDMS with 2% Ninhydrin Reagent at 70 °C for 90 min, followed by measuring the absorbance at 570 nm. The correlation of free amine group within a concentration range of polydopamine solution was also determined with ninhydrin assay.

### Microfluidic chip fabrication

The photomask for chip fabrication was designed with AUTOCAD 2012 and printed on high-resolution transparency films (CAD/Art Services Inc., Oregon, US) which was later used in contact photolithography to produce a master on a 4-inch silicon wafer. In photolithography, SU8-2050 photoresist was spin-coated at 1500 rpm to create an evenly spread film, soft baked to evaporate the solvent and densified before being covered by the photomask and exposed to highly collimated UV light. After UV exposure, the SU-8 was further subjected to post exposure baking to selectively cross-link the exposed portion of the photoresist. The non-exposed portion of the film was etched away by immersing in a developer solution. PDMS microfluidic chip was fabricated by soft lithography whereby ten parts of silicone elastomer base was mixed to one part of curing agents, poured over the master, degassed by vacuum and cured at 70 °C for 2 h before the solidified PDMS was peeled off from the master. The PDMS microfluidic chip was plasma-treated prior to bonding with the uncoated and PD-coated PDMS. Bonded microfluidic chips were UV sterilized before BMSC seeding.

### Osteogenic and adipogenic differentiation

3000 cells per cm^2^ were seeded and cultured for 1 day in expansion medium before being subjected to osteogenic or adipogenic differentiation. For osteogenic differentiation, BMSCs were cultured in osteogenic medium comprising low glucose DMEM, 10% FBS, 1 mM sodium pyruvate, 50 μg ml^-1^ ascorbic acid, 1X glutamax, 100 U/100 μg penicillin/streptomycin, 10 mM β-glycerophosphate, and 10^−7^ M dexamethasone. For adipogenic differentiation, BMSCs were cultured in adipogenic medium comprising high glucose DMEM, 10% FBS, 0.01 mg ml^−1^ Insulin, 0.5 mM IBMX, 0.2 mM Indomethacin, 10^−6^ Dex, 1X glutamax and 100 U/100 μg penicillin/streptomycin. All BMSCs were cultured at 37 °C in humidified atmosphere with 5% CO_2_ with the differentiation medium changed every 2-3 days.

### Gene expression

Differentiated BMSCs after two weeks of osteogenic or adipogenic differentiation were collected for specific differentiated lineage gene expression. The total RNA was harvested with PureLink (R) RNA Mini Kit, and its concentration was quantified with Nanodrop ND2000 (Thermo Scientific, Delaware, USA). 100 ng of total RNA was reverse transcripted with iScript™ Reverse Transcription Supermix kit (Biorad Laboratories, Singapore). mRNA expression level of α5-integrin, β1-integrin, PI3KCG, osteogenesis- and adipogenesis-associated gene markers were quantified by real time polymerase chain reaction (PCR) assays with SYBR PCR Master Mix Kit (Life Technologies, Singapore) and StepOnePlus™ Real Time PCR Systems (Life Technologies, Singapore). The primers used to quantify the specific gene expression were alkaline phosphatase (ALP) and collagen type 1 (Col1) for osteogenic differentiated cells, and Leptin for adipogenic differentiated cells (Table S1). The real-time PCR was initiated at 95 °C for 10 min, followed by 40-cycle amplification that included a denaturation step at 95 °C for 15 s, and an extension step at 60 °C for 1 min. All data were normalized to the GAPDH mRNA level, and later expressed as the mRNA relative change with reference to the BMSCs prior to differentiation using the Livak method.

### Histological staining

The differentiated BMSCs were fixed with 10% formalin overnight prior to histological staining. For the assessment of osteogenesis on different surfaces, fixed BMSCs were immersed in Alizarin red solution for 5 min followed by gentle washing with distilled water until nonspecific staining was removed. For the assessment of adipogenesis on different surfaces, fixed BMSCs were washed with 70% ethanol, stained with Oil Red O solution and counterstained with Haematoxylin. All microscopic images were captured with an Olympus IX71 inverted microscope (Olympus, Singapore). All macroscopic images were captured with a Canon 50D DSLR Camera (Canon, Singapore).

### Statistical analysis

Statistical analysis of all quantitative data was performed by one way analysis of variance (ANOVA), while pairwise comparison of data was determined by Tukey’s HSD (Honestly Significant Difference) test. Statistical significance was set at p-value < 0.05.

## Additional Information

**How to cite this article**: Chuah, Y. J. *et al*. Simple surface engineering of polydimethylsiloxane with polydopamine for stabilized mesenchymal stem cell adhesion and multipotency. *Sci. Rep*. **5**, 18162; doi: 10.1038/srep18162 (2015).

## Supplementary Material

Supplementary Information

## Figures and Tables

**Figure 1:**
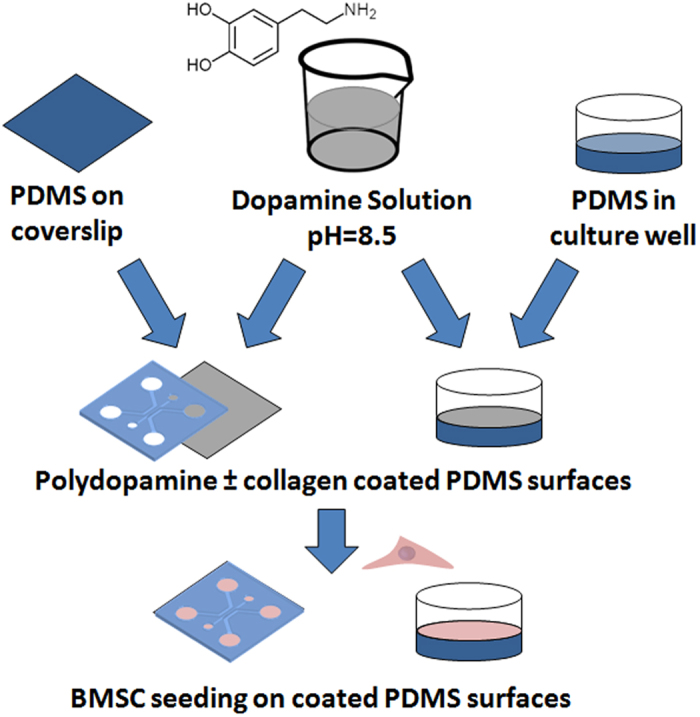
Experimental outline illustrating the functionalization of PDMS surfaces polydopamine to assess prolong BMSCs stability within two culture systems.
