# Supplementary material for: Simple surface engineering of polydimethylsiloxane with polydopamine for stabilized mesenchymal stem cell adhesion and multipotency
Source: Sci Rep. 2015 Dec 9;5:18162. doi: 10.1038/srep18162 (PMC4673458; doi:10.1038/srep18162)
Supplement: Supplementary Information [file srep18162-s1.pdf]

Supplementary Information

Simple surface engineering of polydimethylsiloxane with polydopamine for stabilized mesenchymal stem cell adhesion and multipotency

Yon Jin Chuah[1], Yi Ting Koh[1], Kaiyang Lim[1], Nishanth V. Menon[1], Yingnan Wu[1], and Yuejun Kang[1,*]

[1]School of Chemical and Biomedical Engineering, Nanyang Technological University, 62 Nanyang Drive, Singapore 637459, Singapore

* Email: yuejun.kang@ntu.edu.sg

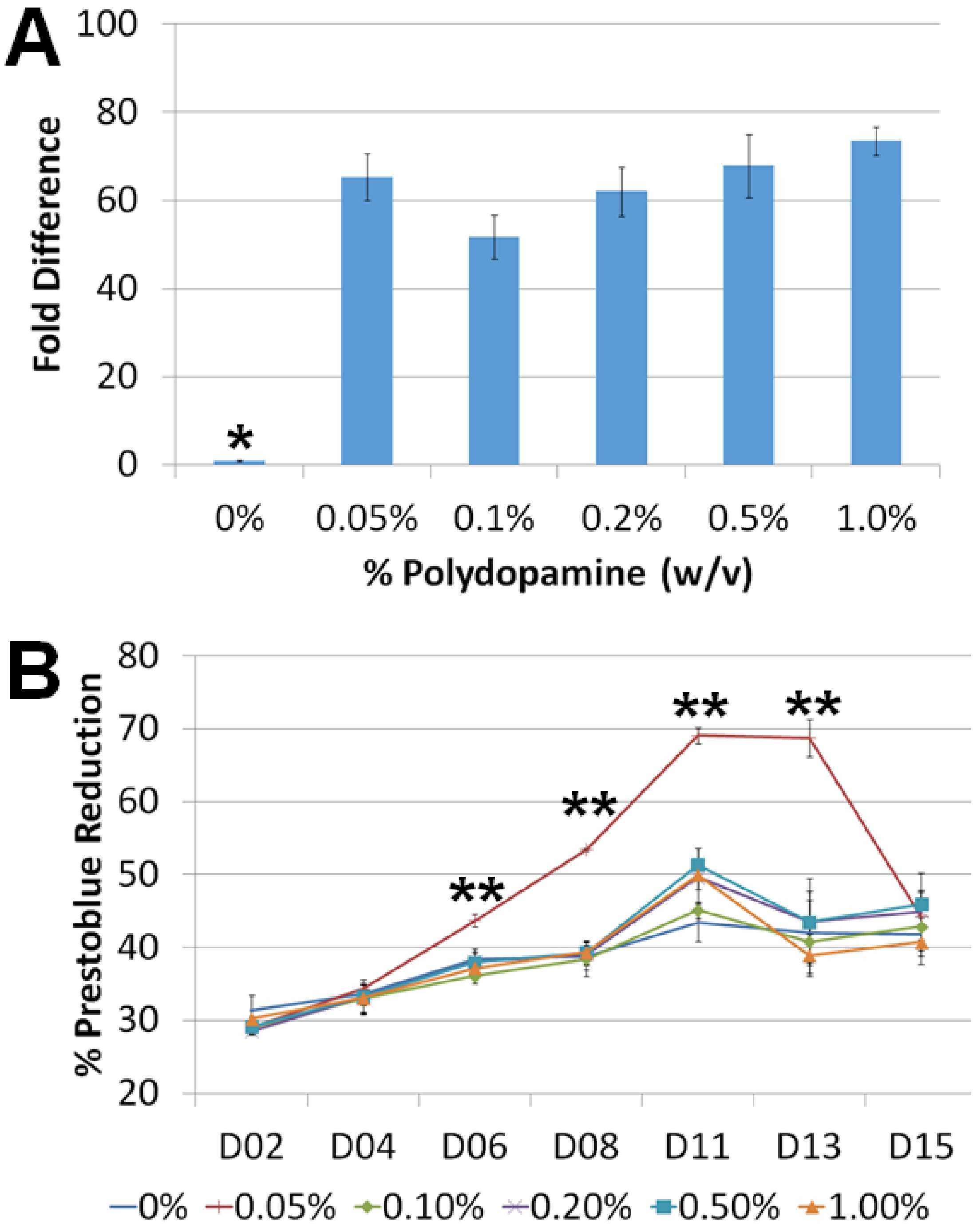

Supplementary Figure S1. Effect of polydopamine concentration (0-1.000% w/v) on BMSC (A) initial adhesion and (B) proliferation over 2 weeks. *p-value = 0.001 shows significant differences as compare to the rest (Tukey HSD test, n = 4). **p-value < 3.61×10^{-6} indicates significant difference between the groups (One way ANOVA, n = 4).

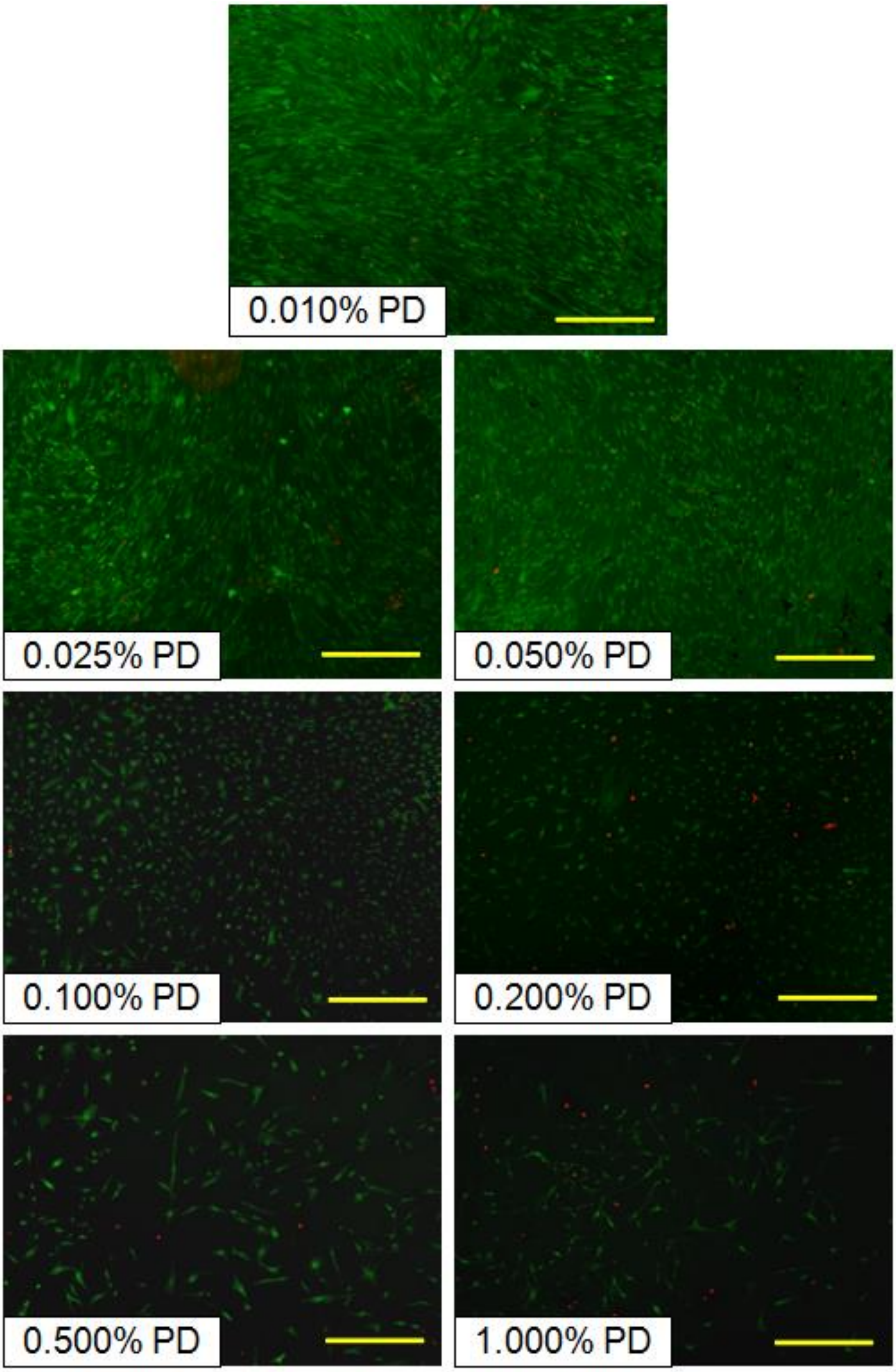

Supplementary Figure S2. Live/Dead Staining of BMSC after two weeks of culture on PDMS substrates with different PD coating concentration (%w/v). Living cells were labelled green and dead cells red. Scale bar = 500 µm.

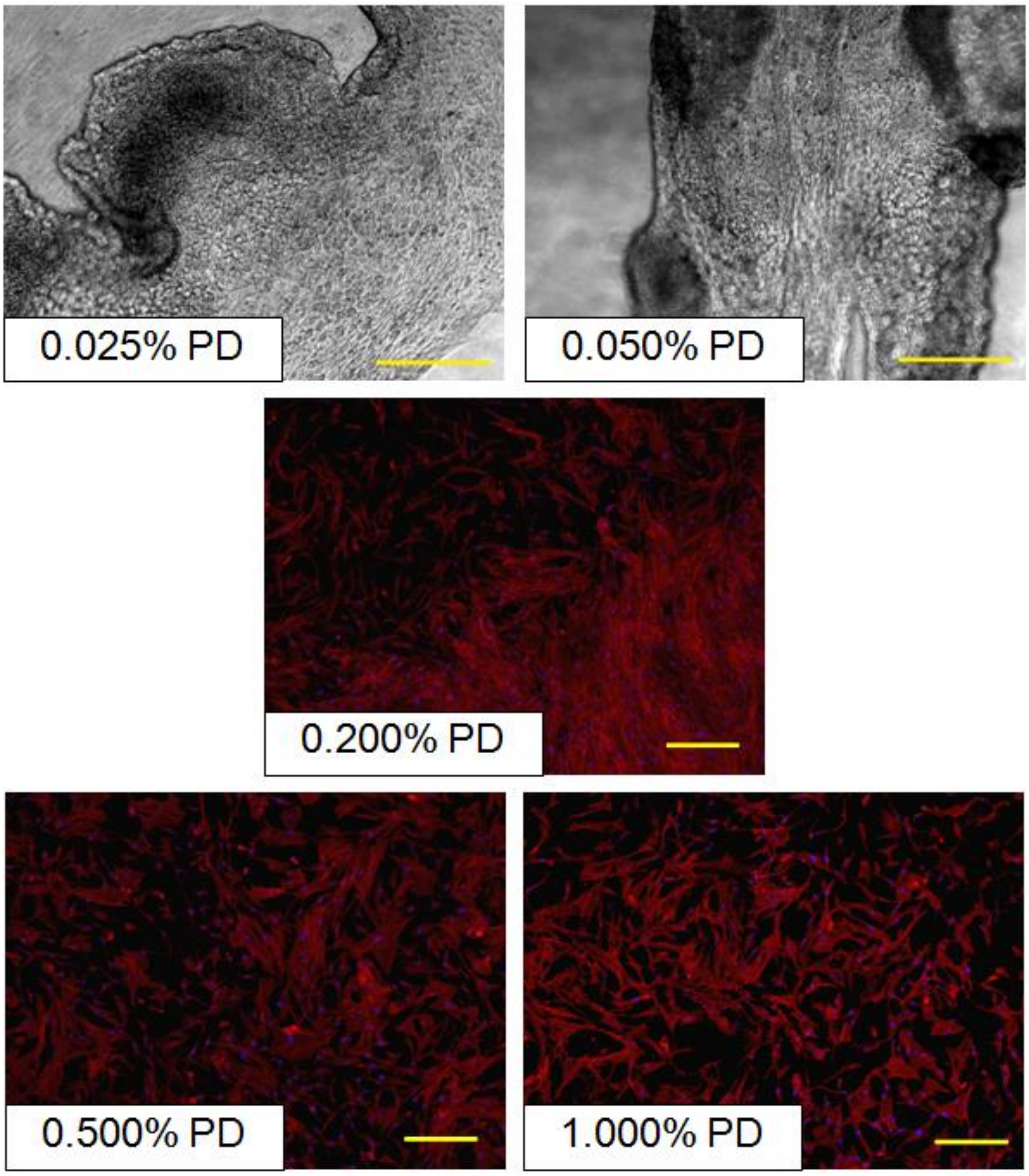

Supplementary Figure S3. Top: BMSC population on PDMS coated with 0.025% and 0.050% PD showing sites of cell aggregation and peeling upon confluence (Phase contrast images, scale bar = 500 μm). Bottom: F-Actin staining with Rhodamine Phalloidin (red) and DAPI (blue) of BMSC population on PDMS coated with 0.2 – 1.0% PD, scale bar = 200 μm.

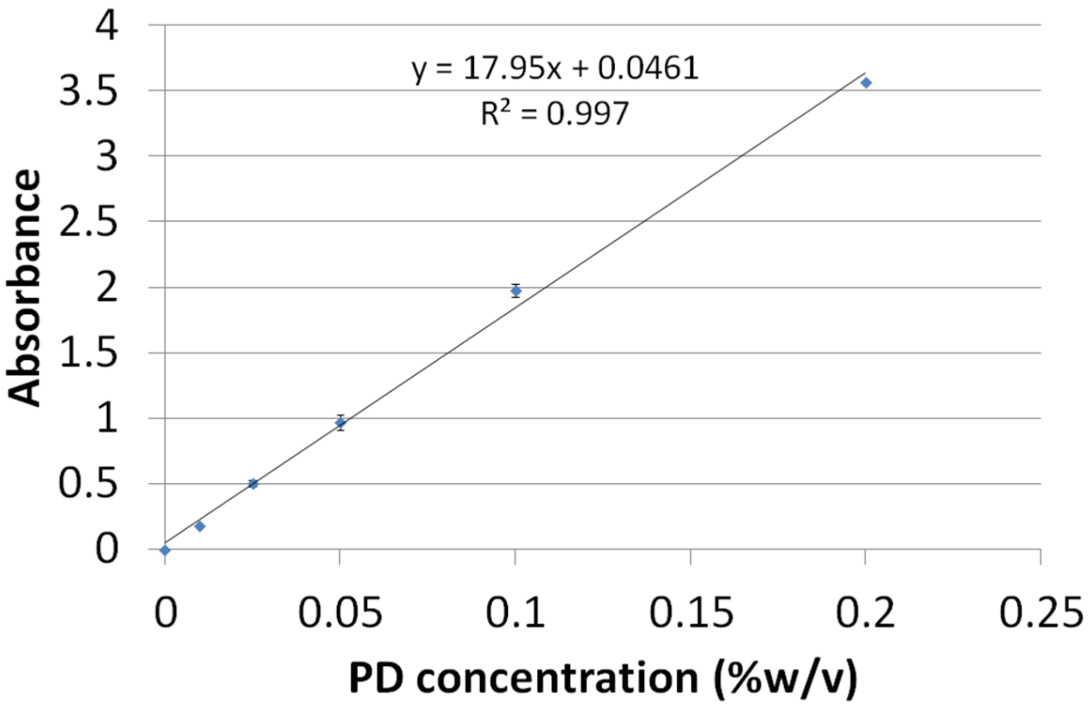

Supplementary Figure S4. Standard curve of PD concentration (% w/v) against absorbance.

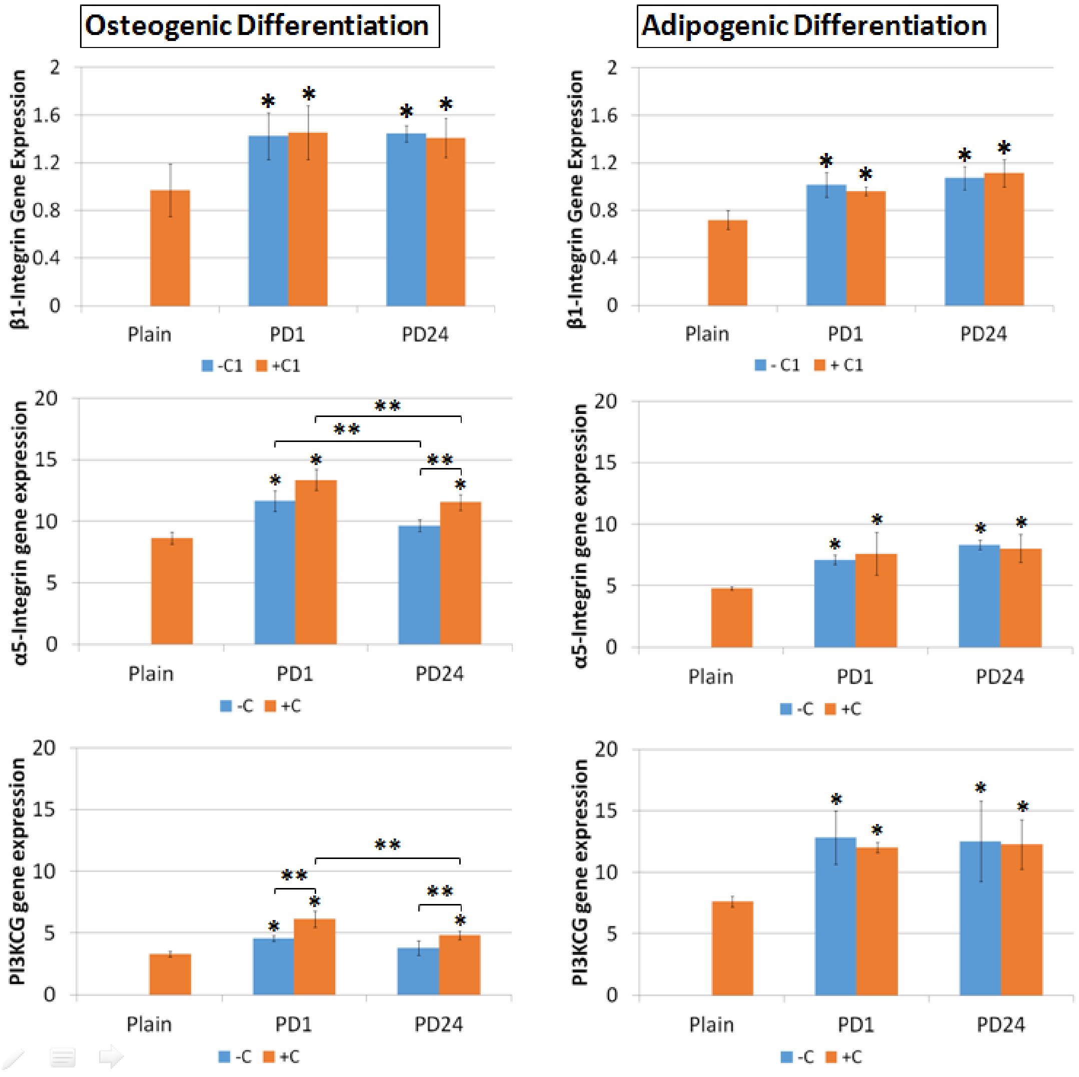

Supplementary Figure S5. Gene expression of β1-integrin, α5-integrin and PI3KCG marker in both osteogenic and adipogenic differentiated MSCs on various PDMS substrates. *p-value < 0.0490 shows significant difference as compared to plain (Tukey HSD test). **p-value < 0.0436 shows significant difference as compared between two groups (Tukey HSD test). All data are presented as mean ± s.d. (n=4).

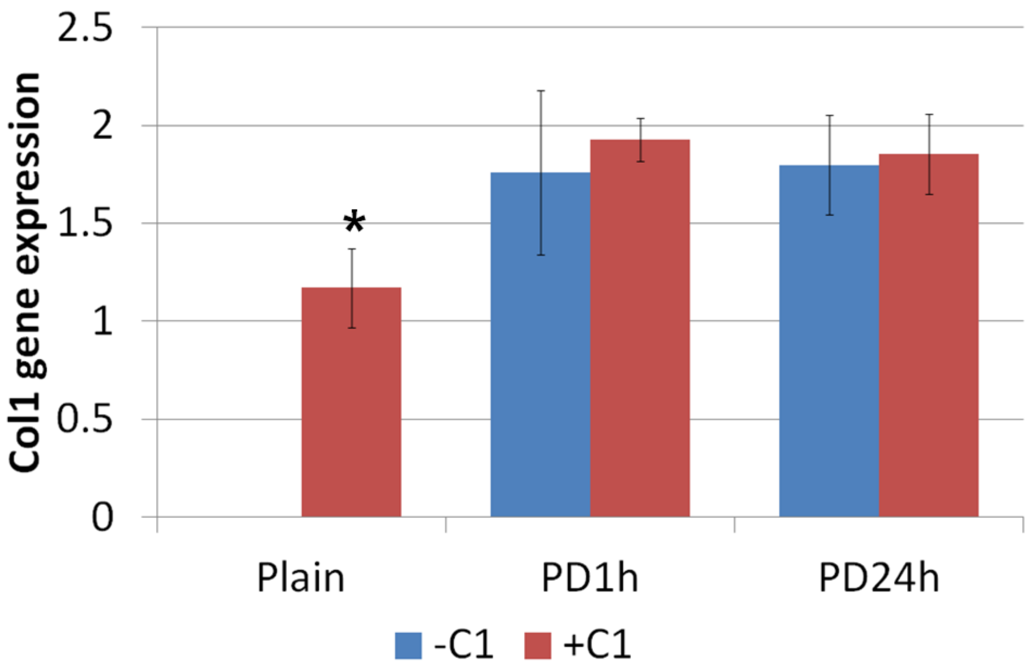

Supplementary Figure S6. Gene expression of osteogenic (Col1) markers. *p-value < 0.0486 shows significant difference as compared to any other group (Tukey HSD test, n = 4).

Table S1. Primer sequences in real time PCR assays.

Gene	Accession Number	Primer Sequence (both 5' - 3')	Product Size
α5-integrin	NM_002205.2	F: GGCTTCAACTTAGACGCGGAG<br>R: TGGCTGGTATTAGCCTTGGGT	63
β1-integrin	NM_033668.2	F: CCTACTTCTGCACGATGTGATG<br>R: CCTTTGCTACGGTTGGTTACATT	128
Collagen Type 1 Alpha 1 (COL1A1)	NM_000088.3	F: CAGCCGCTTCACCTACAGC<br>R: TTTTGTATTCAATCACTGTCTTGCC	83
Alkaline Phosphatase (ALP)	NM_000478.4	F: ACCACCACGAGAGTGAACCA<br>R: CGTTGTCTGAGTACCAGTCCC	79
Leptin	NM_000230.2	F: TGCCTTCCAGAAACGTGATCC<br>R: CTCTGTGGAGTAGCCTGAAGC	164
Phosphoinositide-3-kinase, catalytic, gamma polypeptide (PI3KCG)	NM_002649.3	F: AACACCGACCTCACAGTTTTT<br>R: CTCAAGCCACACATTCCACAG	120
Glyceraldehyde 3-phosphate dehydrogenase (GADPH)	NM_002046.5	F: ATGGGGAAGGTGAAGGTCG<br>R: TAAAAGCAGCCCTGGTGACC	70
